# Evaluation under real-life conditions of a lifestyle intervention for diabetes prevention developed by the municipal health services of Madrid, Spain

**DOI:** 10.1038/s41598-022-21531-7

**Published:** 2022-11-16

**Authors:** Carmen Martin-Ridaura, Darío Ochoa-Esteban, Carmen Berlinches-Zapero, Dolores Ruiz-Fernández, Vanessa Sanz-Martín, Rosario Gavira-Izquierdo, Sebastià March, María López-Toribio, Mercedes Ceinos-Arcones, The ALAS Project Working Group, Dolores Rivas-Hernández, Saray Pino-Vega, Juan Manuel Melero-Rubio, Francisco Bordel-Nieto, Milagros Caballero-Jauregui, Isabel Corella-Monzon, Raquel Pino-Días, Carmen Cutanda-Rodriguez, Sergio Sánchez-Muñoz, José Manuel Fernández-Garrido, Carmen Morales-López, María Jesús Majarrez-Arias, Santiago Sancho, Nuria Calle Fernández, Mercedes Martínez-Cortes, Pilar García-Crespo, Carmen María León-Dominguez, Marina Pascual, Raquel Herrera

**Affiliations:** 1 Diabetes Unit of Madrid Salud, Madrid, Spain; 2 Madrid Salud, Madrid City Council, 62 Mediterraneo Avenue, Floor 6, Madrid, Spain; 3 Cooperativa APLICA, Madrid, Spain; 4 Madrid City Council, Madrid, Spain

**Keywords:** Metabolic disorders, Disease prevention, Patient education, Public health

## Abstract

The Diet, Physical Activity and Health (Alimentación, Actividad física y Salud, ALAS) program is an intervention implemented by the municipal health services of Madrid with the objective of reducing weight and preventing diabetes in high-risk population by improving diet and physical activity. The ALAS program combines individual visits with a 10-session group workshop that takes place over a 6-month period*.* This study evaluated the effectiveness of the ALAS intervention implemented under real-life conditions between 2016 and 2019. The intervention was evaluated with a pre- and post-intervention study with follow-up performed 6 and 12 months from the start of the program. The analyzed outcomes were a 5–10% reduction in the initial weight, body mass index (BMI), waist circumference and a change in glycemic status in prediabetic participants. Statistical models were adjusted by sociodemographic variables. The participants were recruited from municipal community health centers or referred by municipal occupational health services. Between 2016 and 2019, 1629 people participated in the program. At 6 months, 85% of the participants had lost weight; 43% had lost 5% or more of their initial weight, and 12% had lost 10% or more. Regarding BMI, 22.3% of participants who were initially obese were no longer obese, and 15.2% of the overweight participants achieved normal weight. A total of 35.1% of the prediabetic participants reverted to normoglycemic status. The intervention was found to be more effective for men, for those who completed the intervention and those who accessed the program through the occupational health route. Among the participants who accessed the intervention via the community, the intervention was more effective in those with a high educational level. The evaluation demonstrated the effectiveness of the ALAS program for reducing weight and the risk of developing Type 2 diabetes when applied under real-life conditions. The effectiveness of the intervention differed according to gender, access route and educational level of the participants.

## Background

Worldwide, the prevalence of Type 2 diabetes has increased steadily in the last 20 years, and this trend is expected to continue^1^. It is estimated that 13.8% of people in Spain have this disease^2^. In the Madrid region, the prevalence of Type 2 diabetes has been estimated at 9.3%^3^. This prevalence is higher in men than in women, at 12.3% and 6.4%, respectively^3^. In addition, an estimated 63.7% of the adult population in Madrid is overweight^3^. Consequently, the prevention of diabetes and obesity has become a key public health priority in the region.

The development of Type 2 diabetes is preceded by an intermediate period of prediabetes. This state is characterized by higher than normal glucose levels that do not yet meet the diagnostic criteria for diabetes, and it has itself been associated with the onset of cardiovascular complications^4^. The incidence of diabetes is related to metabolic risk factors such as obesity, a sedentary lifestyle, insulin resistance and an increased waist circumference^5^. For this reason, diabetes prevention programs have often focused on lifestyle changes^6^, since the relationship between the development of diabetes and modifiable behaviors such as a lack of physical exercise or an unhealthy diet have been consistently established^7,8^. Additionally, in Spain, the prevalence of known Type 2 diabetes is higher among the population with a lower socioeconomic status^9^ and a lower educational level^10^. These identified social inequalities can be explained in part by the unequal distribution of metabolic and behavioral risk factors^11^.

The city of Madrid has joined more than 39 cities worldwide in Cities Changing Diabetes, a program that seeks to identify effective interventions for the prevention of diabetes in urban contexts^12^. Madrid Salud, the public health agency of the city of Madrid, designed and implemented the Diet, Physical Activity and Health program (Alimentación, Actividad física y Salud, ALAS)^13^ with the aim of promoting healthier lifestyles, reducing obesity and preventing the development of Type 2 diabetes in the population of Madrid. The program includes a strategy for the high-risk population, with educational and structured group workshops on physical activity and healthy eating.

Interventions based on diet modifications and increases in physical activity have been shown to reduce or delay the development of Type 2 diabetes among people with a high risk of developing diabetes^14,15^. In addition, these interventions have proven to be cost-effective from the point of view of the health system and society^16^. Although the evidence suggests that pragmatic interventions for the prevention of diabetes are effective^17^, there is little evidence on the effectiveness of community preventive interventions implemented under real-life conditions in Spain.

This study aimed to evaluate the effectiveness of the ALAS intervention under real-life conditions for reducing weight, body mass index (BMI) and waist circumference and improving the glycemic status of individuals who participated in the program between 2016 and 2019.

## Methods

### Design

The ALAS program was first implemented in 2011. This study analyzes the outcomes of this intervention from 2016 to 2019. To this end, a pre-post evaluation study of the intervention was performed under real-life conditions. The effectiveness of the intervention was measured by comparing the participants’ anthropometric measures (weight, BMI and waist circumference) and the results of laboratory analyses (glycemia and HbA1c) before the intervention, at 6 months (at end of the intervention) and at the 12-month follow-up.

### Participants and study location

Madrid Salud is a municipal body with health promotion and disease prevention functions in the city of Madrid. It serves a population of approximately 160,000 people (according to the service’s information systems in 2019) and has 16 community health centers distributed throughout the city and a referral center for occupational health.

The participants were invited to participate through the municipal health centers, community events, other social health entities and the occupational health services of the city council. To screen the high-risk population, the Finnish Type 2 Diabetes Risk Score (Findrisk) was used^22^; this instrument has been validated in the Spanish population^18^ and identifies people at high risk of developing diabetes. Subjects with a score greater than 14 were referred for a glycemic study that included fasting blood sugar, oral glucose tolerance test (OGTT) and/or glycosylated hemoglobin (HbA1c) to establish the glycemic category.

People between 35 and 69 years old who met any of the following inclusion criteria were invited to participate: a BMI greater than 30 or between 27 and 29 plus a risky waist circumference (greater than 88 cm in women or greater than 102 cm in men) or the American Diabetes Association (ADA) laboratory criteria for prediabetes^19,20^: Fasting baseline glucose between 100 mg/dl (5.6 mmol/L) and 125 mg/dl (6.9 mmol/L), 2-h postprandial glucose between 140 mg/dl (7.8 mmol/L) and 199 mg/dl (11.0 mmol/L) or HbA1c between 5.7% (39 mmol/mol) and 6.4% (46 mmol/mol).

### Description of the intervention

The high-risk strategy of the ALAS program, based on the Diabetes Prevention Program^21^, consists of a community preventive program aimed at reducing weight, improving adherence to the Mediterranean diet pattern and promoting physical activity. It is an intensive intervention that combines individual visits and a structured group education workshop composed of 10 face-to-face 2-h sessions with approximately 15 participants that are implemented over a 6-month period^13^.

The methods used in the group workshop are based on motivation, group support and positive feedback. The program includes the provision of information, group discussions, participants’ recording of their behavior, goal setting and planning. The main topics addressed in the sessions are (1) a healthy and balanced diet, (2) healthy physical activity, (3) thoughts and emotions that favor change, (4) shopping and labeling, (5) cooking techniques, (6) relapse prevention, (7) maintenance planning, (8) approaching special occasions and healthy menus, and (9) psychological techniques to promote and maintain changes in habits. Participants take an active role and work with a task notebook, in which they incorporate the theoretical contents of the sessions into their daily life. In addition, the participants receive printed materials. The workshops are taught at each of the community centers by health professionals with experience in group interventions.

Before the group workshops begin, each participant completes an individual visit that includes anamnesis and anthropometric measurement, assessment of diet and physical activity, and a blood analysis to measure baseline glycemia and HbA1c and/or OGTT, which are used to classify the participant as normoglycemic, prediabetics or diabetics, according to the ADA classification^19,20^. At 6 and 12 months after starting the program, another individual clinical and analytical assessment is performed to assess the changes in these parameters.

### Variables

The main outcome variables were weight, BMI, waist circumference and glycemic status, which are collected at the individual visits. The goals of the intervention are a 5% to 10% reduction of the initial weight, the downgrading of the initial BMI classification, a smaller waist circumference and a return to normoglycemia among people who were initially classified as having prediabetes.

The collected sociodemographic variables were educational level (primary, high school, university), employment status (working, unemployed, studying, home care, retired/pensioner), origin (born in Spain or outside of Spain) and marital status (single, married, separated/divorced, widowed).

In addition, whether the intervention had been completed as defined in the program protocol (attendance at least 6 of the 10 sessions) was recorded. The route of recruitment to the program (through municipal occupational health services or the community) was also recorded.

### Statistical analysis

A stratified descriptive analysis was performed according to the participants’ route of access to the intervention. Kolmogorov–Smirnov normality tests were performed on continuous variables to adapt the hypothesis contrast tests to be used. The chi square and paired Student’s t tests were used to compare categorical or quantitative variables, respectively, between the groups and to analyze the effectiveness of the intervention according to changes in the variables at 6 months (at the end of the intervention) and 12 months (at the postintervention follow-up). The effectiveness results were also stratified by sex and access route.

To examine the factors related to the effectiveness of the intervention, 3 logistic regression models were performed using a 5% reduction in baseline weight as the dependent variable: one model included all participants, another included only those who had accessed the program via occupational health, and the last one included only those who had accessed the program through the community. The analyses were performed for the portion of the sample with complete data for all the variables used in the model. The presented models include the variables that showed a statistically significant relationship in the bivariate test with the dependent variable. The unadjusted proportions of the main variable and the adjusted ORs and corresponding confidence intervals for the remaining variables are presented. Statistical analyses were performed with SPSS version 18 software.

### Ethical aspects

The ALAS intervention has been implemented for more than ten years in the city of Madrid. The intervention was considered a routine practice of Madrid Salud. All participants took part in the intervention voluntarily. Madrid Salud gave its approval and support to evaluate the intervention according to the methodology described above. The data used in this study were those collected routinely during the implementation of the intervention. The owner institution of the data is Madrid Salud. The data were provided fully anonymized to the external analysts. The study complied with the principles of the Declaration of Helsinki of 2013^22^.

## Results

From March 2016 to July 2019, baseline information was collected from 1629 participants in the ALAS program, of whom 1219 (75%) were women. At the end of the intervention, the information of 1021 people (62.7%) was recorded, and at 12 months after the start of the intervention, the information of 150 people (9.2%) was recorded. The number of participants with complete information for each outcome variable and temporal cutoff is presented in Table 1.

**Table 1:** Complete values for the outcome variables at each time point.

	Start of intervention	End of intervention	Follow-up 1 year after start
	N (% total sample)	N (% total sample)	N (% total sample)
Sample	1.629 (100%)	1.021 (62.7%)	150 (9.2%)
Weight	1.628 (99.9%)	1.021 (62.7%)	150 (9.2%)
Waist circumference	1.486 (91.2%)	617 (37.9%)	148 (9.1%)
Glycemic status	1.165 (71.5%)	414 (25.4%)	60 (3.7%)
BMI (height and weight)	1.595 (97.9%)	1.010 (62.0%)	150 (9.2%)

A total of 31% (n = 501) of the participants accessed the intervention through the municipal occupational health service. The baseline description of the participants according to their route of access to the intervention is shown in Table 2. Except for the BMI and the waist circumference, the remaining variables differed significantly between the individuals referred by municipal occupational health services and those who accessed the program via the community. Among the participants who were referred by occupational health services, there was a significantly larger population with prediabetes, more men, younger people, more people born in Spain, more married people and more people with a university education.

**Table 2:** Baseline description of the sample according to the access route to the intervention.

Qualitative variables		Total	Access route		P*
			Occupational health	Community	
		N (% column)	N (% column)	N (% column)	
Total		1629 (100)	501(31)	1128 (69)	
Body mass index	Normal weight	54 (3)	12 (3)	42 (4)	0.384
	Overweight	482 (30)	151 (31)	331 (30)	
	Obesity	1059 (66)	320 (66)	739 (66)	
Glycemic status	Normoglycemic	689 (59)	240 (51)	449 (65)	< 0.001
	High risk for diabetes	428 (37)	218 (47)	210 (30)	
	Diabetic	48 (4)	11 (2)	37 (5)	
Waist circumference risk	Yes	1268 (86)	384 (83)	884 (87)	0.034
	No	210 (14)	79 (17)	131 (13)	
Sex	Male	401 (25)	197 (39)	204 (18)	< 0.001
	Female	1219 (75)	303 (61)	916 (82)	
Age	< 44 years	228 (15)	56 (12)	172 (16)	< 0.001
	45–54 years	458 (30)	205 (44)	253 (23)	
	55–64 years	470 (30)	164 (36)	306 (28)	
	> 65 years	389 (25)	36 (8)	353 (33)	
Nationality	Spanish	1400 (92)	459 (99)	941 (89)	< 0.001
	Not Spanish	118 (8)	3 (0,6)	115 (11)	
Marital status	Single	252 (17)	58 (13)	194 (19)	< 0.001
	Married	948 (65)	335 (74)	613 (61)	
	Separated	164 (11)	48 (10)	116 (12)	
	Widowed	93 (6)	14 (3)	79 (8)	
Work situation	Working	789 (55)	414 (91)	384 (39)	< 0.001
	Unemployed	140 (10)	3 (0.7)	137 (14)	
	Studying	11 (1)	1 (0.2)	10 (1)	
	Home care	160 (11)	6 (1.3)	154 (15)	
	Retired/pensioner	340 (23)	33 (7.2)	307 (31)	
Education level	Primary or less	246 (17)	22 (5)	224 (23)	< 0.001
	Secondary	723 (51)	194 (43)	529 (54)	
	University	462 (32)	237 (52)	225 (23)	

Quantitative variables	Mean (SD)	Mean (SD)	Mean (SD)	p^†^	
Age (years)	56.1 (11.26)	53.5 (7.67)	57.2 (12.32)	< 0.001	
Weight (kg)	84.8 (15.48)	88.5 (15.49)	83.2 (15.21)	< 0.001	
Height (cm)	163 (9.12)	166 (9.11)	161 (8.63)	< 0.001	
Body mass index	31.9 (4.81)	31.9 (4.53)	32.0 (4.93)	0.756	
Waist circumference (cm)	103.8 (12.39)	104.5 (11.99)	103.5 (12.55)	0.113	

*p-value for the chi-squared test of the relationship with the access route. p < 0.05 implies a significant relationship.

^†^p-value for Student’s t test for the comparison of means according to the route of access to the intervention.

### Effectiveness of the intervention

Table 3 shows the measurement of the intervention’s goals at its completion. At 6 months, the participants had lost an average of 4.3 kg (SD = 4.6), which represented an average of 4.96% (SD = 4.8) of their baseline weight; additionally, they had a 4.2 cm (SD = 5.2) reduction in waist circumference and a 1.6 kg/m^2^ (SD = 1.6) decrease in BMI. Eighty-five percent of the participants reduced their weight by at least 1 kg, 43% of the participants lost at least 5% of their baseline weight, and 12% reduced their initial weight by 10% or more. In addition, 79.2% decreased their waist circumference by at least 1 cm. At the end of the intervention, 74.3% of the participants remained with a high-risk waist circumference (more than 88 cm in women or more than 102 cm in men), which represents a reduction of 12.5% with respect to the baseline measurement (95% CI = 9.5–14.8%). A total of 19.3% of the participants downgraded their BMI classification: Among those initially classified as obese, 22.3% were no longer obese (21.8% reverted to overweight, and 0.5% reverted to normal weight), and among those who were overweight, 15.2% reverted to normal weight. With respect to glycemic status, 35.1% of the people who were initially classified as prediabetics were normoglycemic at the end of the intervention. All of these results showed statistically significant associations with the route of access to the intervention and participant sex, which were confirmed with Student’s t test for pre-post quantitative changes or the chi-square test for the qualitative goals (p < 0.05). For all goals, the intervention was more effective for people who accessed the program via the occupational health route than for those who accessed it via the community and was more successful for men than for women.

**Table 3:** Outcomes at the end of the intervention by sex and access route.

Quantitative outcomes		All participants		Occupational health access		Community access	
		N*	Change^†^ Mean (95% CI)	N*	Change^†^ Mean (95% CI)	N*	Change^†^ Mean (CI95%)
Weight	All	1020	4.3 (4.1–4.6)	450	5.5 (5.0–6.0)	570	3.4 (3.1–3.7)
	Males	275	6.1 (5.4–6.8)	176	7.0 (6.1–8.0)	99	4.5 (3.5–5.5)
	Females	741	3.7 (3.4–3.9)	273	4.5 (4.0–5.1)	468	3.2 (2.9–3.4)
Waist circumference	All	610	4.2 (3.8–4.6)	205	6.0 (5.3–6.8)	405	3.3 (2.8–3.7)
	Males	155	6.0 (5.1–6.9)	84	7.3 (6.0–8.6)	71	4.5 (3.4–5.6)
	Females	453	3.6 (3.1–4.0)	120	5.2 (4.2–6.1)	333	3.0 (2.5–3.5)
Body mass index	All	1010	1.6 (1.5–1.7)	440	2.0 (1.8–2.2)	570	1.3 (1.2–1.4)
	Males	279	2.0 (1.8–2.2)	174	2.4 (2.1–2.7)	105	1.4 (1.1–1.7)
	Females	727	1.5 (1.4–1.6)	265	1.8 (1.6–2.0)	462	1.3 (1.2–1.4)

Qualitative outcomes		N*	Success % (95% CI)	N*	Success % (95% CI)	N *	Success % (CI 95%)
5% reduction in baseline weight	All	1020	43.0 (40.0–46.1)	450	51.1 (46.5–55.7)	570	36.7 (32.7–40.6)
	Males	275	53.1 (47.2–59.0)	176	57.9 (50.6–65.3)	99	44.4 (34.5–54.4)
	Females	741	39.4 (35.9–42.3)	273	46.9 (40.9–52.8)	468	35.0 (30.7–39.4)
Smaller waist circumference	All	610	79.2 (75.9–82.4)	205	87.3 (82.7–91.9)	405	75.1 (70.8–79.3)
	Males	155	88.4 (83.3–93.5)	84	92.8 (87.2–98.5)	71	83.1 (74.2–92.0)
	Females	453	75.9 (72.0–79.9)	120	83.3 (76.6–90.1)	333	73.3 (68.5–78.0)
Lower BMI classification	All	1010	19.3 (16.9–21.7)	440	22.5 (18.6–26.4)	570	16.8 (13.8–19.9)
	Males	279	26.5 (21.3–31.7)	174	27.6 (20.9–34.3)	105	24.8 (16.4–33.2)
	Females	727	16.6 (13.9–19.4)	265	19.2 (14.5–24.0)	462	15.1 (11.9–18.4)
Initially obese participants who are no longer obese	All	660	22.3 (19.1–25.5)	295	26.1 (21.1–31.1)	365	19.2 (15.1–23.2)
	Males	207	30.4 (24.1–36.8)	124	34.7 (26.2–43.2)	83	24.1 (14.7–33.5)
	Females	450	18.7 (15.0–22.3)	170	20.0 (13.9–26.1)	280	17.9 (13.3–22.4)
Initially overweight participants who revert to normal weight	All	315	15.2 (11.2–19.2)	135	16.3 (10.0–22.6))	180	14.4 (9.3–19.6)
	Males	69	15.9 (7.1–24.8)	48	10.4 (1.4–19.4)	21	28.6 (7.5–48.6)
	Females	246	15.4 (10.5–19.5)	87	14.4 (9.3–19.6)	159	12.6 (7.4–17.8)
High risk participants who revert to normoglycemia	All	188	35.1 (28.2–42.0)	125	44.0 (35.2–52.8)	63	17.5 (7.8–27.1)
	Males	69	47.8 (35.7–59.9)	56	51.8 (38.3–65.3)	13	30.8 (1.7–59.8)
	Females	119	27.7 (19.6–35.9)	69	37.7 (25.9–49.4)	50	14.0 (4.0–24.0)

*Sample size with complete data for the calculation of the parameter.

^†^All the changes are negative, and all Student’s t tests for the pre-postintervention difference of means had a p-value < 0.01.

### Factors related to effectiveness

Table 4 shows the adjusted ORs of the variables that had a statistically significant relationship with the main goal of achieving a 5% reduction in baseline weight in the bivariate (chi-square) test. For the model that included all participants, the factors that had a positive influence after adjustment for the rest of the variables were male sex, having completed the entire intervention (6 or more of the 10 sessions) and accessing the program through the municipal occupational health service. For the model that included only the participants who accessed the intervention via occupational health services, the factors that maintained the effect after adjustment were male sex and completing the entire intervention. On the other hand, in the community model, the factor with the greatest influence on the effectiveness of the intervention after adjustment was education level: after adjustment for the rest of the factors, the intervention was 70% more effective in participants with a university education than in others.

**Table 4:** Proportions and adjusted ORs for a 5% reduction in weight by access route.

	All model^‡^ N = 757		Occupational health access model^§^ N = 326		Community access model^||^ N = 431	
	%*	Adjusted OR^†^ (95% CI)	%*	Adjusted OR^†^ (95% CI)	%*	Adjusted OR^†^ (95% CI)
**Sex**						
Male	53.1	**1.79 (1.26–2.54)**	58	**2.24 (1.39–3.6)**	44.4	1.45 (0.85–2.46)
Female	39.4	Reference	46.9	Reference	35.0	Reference
**Age**						
Younger than 55 years	47.6	1.30 (0.94–1.80)	53.5	1.24 (0.77–2.01)	40.4	1.38 (0.88–2.16)
55 years or older	38.4	Reference	47.2	Reference	34.0	Reference
**University studies**						
No	39.7	Reference	53.9	Reference	32.6	Reference
Yes	50.1	1.22 (0.88–1.69)	51.6	0.84 (0.52–1.33)	47.6	**1.70 (1.06–2.70**)
**Working condition**						
Currently not working	35.6	Reference	53.7	Reference	33.3	Reference
Working	48.7	1.22 (0.78–1.66)	52.0	0.80 (0.37–1.75)	42.2	1.11 (0.71–1.74)
**Baseline BMI classification**						
Normal range	25.7	Reference	30.0	Reference	24.0	Reference
Overweight	42.5	2.01 (0.75–5.37)	51.1	2.17 (0.37–12.6)	36.1	1.98 (0.60–6.54)
Obese	45.3	2.19 (0.83–5.77)	53.8	2.56 (0.45–14.48)	38.3	2.15 (0.66–6.97)
**Intervention completed**						
No	34.5	Reference	35.4	Reference	33.3	Reference
Yes	47.7	**2.1 (1.40–3.14)**	58.3	**2.67 (1.58–4.58)**	40.0	1.70 (0.90–3.19)
**Access to intervention**						
Community	36.7	Reference				
Occupational health	51.1	**1.61 (1.12–2.33)**				

*Prevalence of success in reducing the baseline weight by 5%, without adjustment.

^†^Bold type indicates the values whose input into the model yielded results that were significant at p < 0.05.

^‡^Nagelkerke’s R square 0.096.

^§^Nagelkerke’s R square 0.108.

^||^Nagelkerke’s R square 0.050.

### Effectiveness at 1 year after the start of the intervention

The participants with complete data one year after the start of the intervention were only 9.2% of the initial ones, so these data should be interpreted with caution. The average weight and waist circumference of the participants who were followed for 1 year decreased significantly, indicating that the effect of the intervention persisted in the medium term (Table 5). The same was true for the percentage of people with obesity or a high-risk waist circumference. Among those for whom complete data were available (N = 150), 47.3% (95% CI = 39.2–55.4) achieved the 5% weight loss goal from the start of the intervention to the 1-year follow-up. This was a statistically significant improvement (Student’s t p-value = 0.01) with respect to the 36.7% efficacy of the same population at the end of the intervention. The same was true for the reduction of the BMI classification at the 1-year follow-up (26.7%; 95% CI = 19.5–33.8) compared to the same sample at the end of the intervention (20.8%). Therefore, the intervention’s effectiveness for weight reduction continued after the intervention ended.

**Table 5:** Medium-term intervention outcomes (with complete data at 1 year).

	N (complete data)	Start of the intervention	End of the intervention	Follow-up 1 year after the start of the intervention	p* (baseline to 1 year of follow-up)
Average weight (kg)	147	80.4	77.1	76.6	< 0.01
Mean WC (cm)	144	100.4	99.7	96.7	< 0.01
Average BMI	147	30.7	29.4	29.2	< 0.01
% obese	147	59.2	44.9	42.2	< 0.01
% prediabetic	51	23.4	27.5	17.6	0.261
% WC risk	145	83.8	71.3	69	< 0.01

*p-value of the Student’s t test for paired samples for the means at the start of the program and 1 year after the intervention.

## Discussion

This study evaluated the impact of the ALAS intervention for the prevention of diabetes among the population with obesity or prediabetes in the city of Madrid from 2016 to 2019. The intervention, when implemented under real-life conditions, showed effectiveness for reducing the participants’ weight, initial BMI classification and waist circumference and for changing an initial status of prediabetes to normoglycemia. The effect lasted 12 months after the start of the intervention. The intervention was more effective in men, those who completed the intervention and those who accessed the intervention via occupational health. Among the people who accessed the intervention via the community, the intervention was more effective among those with a university education.

Regarding the effectiveness of the intervention at 6 months, 43% of the participants lost at least 5% of their initial weight, and the average weight loss per person was 4.3 kg (95% CI 4.1–4.6). These results are similar to those of other studies that evaluated lifestyle interventions^23^. The weight loss seemed to be maintained at 12 months, which is consistent with longitudinal studies showing that weight loss would be maintained in the long term^24^. It has been shown that even small weight reductions have positive effects on health, such as decreases in blood pressure or cholesterol concentration, which are known risk factors for cardiovascular diseases^25^. In addition, it has been indicated that a weight loss of at least 5% is associated with a 58% decrease in the risk of developing diabetes in the following 4 years^21^. Regarding glycemic status, 35% of the people who were classified as prediabetic at the start of the intervention were classified as normoglycemic at 6 months. It has been estimated that the risk of developing diabetes decreases by 56% in those who achieve normal blood sugar regulation compared to those who remain at the prediabetic level^26^. Regarding waist circumference, the participants reduced their waist circumference by an average of 4.2 cm (95% CI 3.8–4.6). It has been shown that waist circumference is an independent risk factor for the development of Type 2 diabetes as well as other cardiovascular diseases, suggesting that its reduction could have positive effects on participants’ health in the long term^27^.

The effectiveness of the intervention for weight loss was greater in people who participated in at least 6 of the 10 sessions. This indicates that the threshold of 60% of the sessions that was defined by the program is adequate for determining appropriate adherence to the intervention program. This result can serve to motivate adherence to the program. In addition, it seems to indicate a dose–response effect of the intervention, since the greater the participants’ exposure to the intervention was, the greater their weight loss was. This effect was observed in both subsamples, but it only remained significant in the model that included participants who accessed the program through occupational health.

The intervention was more effective in the group of participants who accessed the program through occupational health. This could be due to several factors. On the one hand, this access route could be related to greater motivation for change, since city council officials may be aware of the possibility of referral to this program and actively seek opportunities to participate, an approach that is less likely in the community environment, where the majority of recruitment is opportunistic^28^. A greater initial motivation for change has been associated with greater success in lifestyle change interventions^29^. The collaborative effort of referring participants from occupational health services to the program may have a positive effect, since the outcomes of this subgroup were better than those reported for other health promotion interventions in the workplace^39^. On the other hand, the group of participants who were referred by the occupational health program included a greater proportion of young people, men, people born in Spain and people with higher education levels. The majority of this population are civil servants or former civil servants; therefore, they generally have greater job stability and purchasing power than the average population. All these variables are associated with a better social position, which has been associated with greater participation in and effectiveness of diabetes prevention interventions^30,31^.

Regarding sex, the intervention was more effective for men than for women in terms of all outcome variables. On average, the men lost 2.4 kg of weight, 2.4 cm of waist circumference and 0.5 kg/m^2^ of BMI more than the women did. Therefore, although the men did not participate as much in the intervention, they benefited more from it than the women did, a fact that has been reported for other weight loss interventions^32^. One possible explanation for this finding is that biologically, men tend to lose weight more easily and more quickly than women^33^. It has been hypothesized that women could benefit more from longer interventions, since they tend to lose weight more slowly than men^34^. In addition, men tend to start interventions with higher rates of obesity and therefore tend to lose weight more easily^32^. Even though the effectiveness model was adjusted for the initial BMI, the differences between the 2 sexes in the general model and the occupational health access route model remained statistically significant. Other possible explanations are that gender was not sufficiently considered in the content or in the moderation of the group sessions, which may have allowed men to participate more, receive more attention and obtain more positive reinforcement than women, as described in other studies^35^.

Among the participants who accessed the program through the community, the intervention was more effective for those with higher education levels. This difference has also been observed in other diabetes prevention interventions^36^. The ALAS intervention includes lessons and techniques for recording one's own behavior that may be more accessible and easier to complete for people with more education. In addition, it has been described that people with a lower level of education may have more difficulties understanding the information provided in health education interventions and applying related changes due to low health literacy^37,38^. These results are consistent with the inverse care law, in which people with a more privileged social position benefit more from health interventions^39^.

### Strengths and limitations

To our knowledge, this is the first evaluation performed under real-life conditions of a Type 2 diabetes prevention intervention that has been implemented for more than 10 years in the city of Madrid. The 1629 individuals who participated in the high-risk intervention portion of the program between 2016 and 2019 indicate the size and scope of this municipal health promotion program. However, the study has certain limitations that must be considered. Because the study evaluated a program that was already being implemented and offered to the entire population of Madrid, it was not ethically possible to establish a study design with a control group. In terms of follow-up, at the end of the intervention, the weight of 62.7% of the baseline participants was collected, while at 12 months from the start of the intervention, weight was collected for only 9.2% of the participants. These losses may be due more to the difficulty of reconnecting with the participants in real life than to their abandonment of the intervention. Even with these losses, the sample size was sufficient to show the effectiveness of the program in the short- and medium-term. However, it is not possible to rule out a follow-up bias, in which people for whom an intervention is more effective tend to remain involved with the intervention^40^. Therefore, greater effort is necessary to ensure participant follow-up of and confirm the long-term effectiveness of the intervention. The stratified analyses imply that the sample was fragmented, which means that statistical power was lost and that the statistical significance of some factors studied cannot be guaranteed. Despite this limitation, a sufficient number of important factors was significant in the adjusted models, and their effect on the effectiveness of the intervention can be ensured. Future studies could use the qualitative methodology to explore the barriers to the participation of the most disadvantaged groups and to examine the role of sex and education level in the effectiveness of the intervention.

## Conclusions

The ALAS program shows short- and possibly medium-term effectiveness for reducing obesity and preventing Type 2 diabetes when applied under real-life conditions in the urban context of the city of Madrid. The effectiveness of the intervention differed according to gender, access route and educational level. More studies are needed to explore the long-term effectiveness of the program and to examine the factors that influence participation in and follow-up with health promotion interventions.

## Data Availability

The datasets used and/or analysed during the current study are available from the corresponding author on reasonable request.

## Abbreviations

BMI: Body mass index
HbA1c: Hemoglobin type A, subfraction 1c (glycated hemoglobin)
OGTT: Oral glucose tolerance test
